# Should Moral Repair Be Offered to Morally Injured Laboratory Animal Technicians?

**DOI:** 10.1111/bioe.70128

**Published:** 2026-05-20

**Authors:** John Goris, Jane Johnson

**Affiliations:** ^1^ Macquarie University Sydney New South Wales Australia

**Keywords:** animal experiments, lab technicians, moral injury, moral repair, Rawls

## Abstract

Lab‐technicians are at risk of sustaining moral injuries when complicit in unethical experiments. Prima facie, it would be puzzling to offer the perpetrator of an unethical experiment psychological support in the form of moral repair. However, we argue that lab technicians are owed moral repair as a special case of our proposed duty of special concern. The duty of special concern states that special consideration must be given to the welfare of those who undertake substantial risks for the benefit of others in a cooperative scheme. We make sense of the substantial risk of moral concern lab technicians face by drawing on Rawls' notion of imperfect procedures of justice. Imperfect procedures of justice are those that aim for just outcomes, but procedures do not guarantee those outcomes. Animal experimentation belongs to this category, as it aims for only ethical experiments to be conducted, yet that result is not guaranteed by its procedures. The risk of moral injury falls heavily on lab technicians as they will be the ones to implement the unethical experiment. Hence, we make sense of the puzzling intuition that lab technicians have conducted an unethical experiment yet are owed psychological support.

## 1 Introduction

The role of a laboratory animal technician (henceforth a ‘lab technician’) directly relates to the care and use of animals in research. Their duties often include attending to the basic physical needs of animals by providing food and water, keeping animal housing clean, and monitoring and recording information about an animal, including taking samples such as blood. At the end of research or if there is excess animal stock, they may perform animal euthanasia.

The work of lab technicians is ethically complex. They often perform work that may be recognised as important, like when research is for the development of medicines [1], yet they can also feel morally stigmatized if they are perceived to harm animals in research [2]. Their work contains an apparent contradiction between caring for animals and inflicting harm and suffering on animals (the so‐called ‘caring‐killing paradox’). Lab technicians must tend to animals to keep them alive and comply with ethical standards regarding their treatment, yet they may also be involved in protocols in which animals suffer, and they may kill animals if required by an experiment. Given this ethical complexity, lab technicians would seem to be at risk of moral injury [2, 3, 4, 5].

Moral injury is a type of harm that occurs when one performs an act that violates a personally held moral principle or value, or witnesses such an act, which results in psychological turmoil [6, 7]. In some cases of moral injury, one must hold that certain acts are morally impermissible yet perform them anyway.^1^ It is not simply a weakness of will that causes one to perform these morally impermissible acts. These acts are often committed in the context of institutional pressures [8].

The phenomenon of moral injury has been the subject of psychological research. Several meta‐analyses and systematic reviews of the psychological literature indicate that those experiencing moral injury suffer ‘severe distress and functional impairment’ that impacts their social relations, religious commitments, and may even have neurobiological outcomes [9]. Hall and colleagues [10] found that moral injury is associated with poor mental health outcomes such as anxiety, depression, suicidality and substance use. These associations may differ depending on occupational context. As (Williamson et al. [11], and McEwan et al. [12]) note, most studies examine cases of moral injury in military personnel. Nevertheless, meta‐analyses of other groups, such as health professionals, also find evidence of a significant relationship between moral injury and poor mental health [13]. In short, moral injury seriously harms one's wellbeing.

Moral repair is the psychological process whereby one overcomes moral injury [7]. Again, there is a psychological literature devoted to studying the most effective methods for effecting moral repair. Treatments for moral injury include Cognitive Processing Therapy [14], self‐compassion [15] and mindfulness [16]. However, not all treatments are post hoc and not all treatments are individual. Some proposed interventions are prophylactic and structural such as providing soldiers with mental health training before deployment [17]. For brevity, we will simply refer to moral repair as all those interventions which aim to both prevent moral injury and address it when it has occurred.

Indeed, much of the literature on moral injury comes from psychology and is motivated to investigate moral injury empirically and identify clinical interventions. However, this practical orientation glosses over the prior normative question as to whether there is a duty to offer moral repair to individuals. After all, to be morally injured, one has to believe that one has done something morally wrong, and in some cases, that belief is correct. It is prima facie strange to suggest that there is a duty to support the perpetrators of immoral actions. Yet we maintain that it is reasonable to hold the intuition that lab technicians have done something immoral (i.e., by conducting an unethical experiment) yet are, in some sense, a “victim”. This is a puzzling intuition, but over the course of our argument, we show it to be plausible and that institutions hold a duty to offer moral repair to lab technicians.

Before continuing we must clarify that there cannot be a duty to supply moral repair just as there is no duty to supply someone good health. Mental health, as with physical health, is not something that can be provided by others. A patient suffering from an incurable chronic illness, for example, cannot claim her doctors are failing in their duty to provide her with good health since it is impossible to dispense this duty. And since we cannot be morally compelled to discharge duties that are impossible, there can be no duty to provide others with good health. What we can provide (and may in some instances have a duty to provide) are the goods and services that help support good health through preventing illness and injury and through treating them when they occur. But to avoid cumbersome sentences like ‘offer mental healthcare services that prevent moral injury and target moral repair’ we will instead use sentences like ‘offer moral repair.’

So, ought lab technicians be offered moral repair?

We will argue that there are two ‘easy’ answers to this question which apply in some rare cases but are unhelpful in the majority of situations. We then draw upon and modify Rawls' notion of imperfect systems of justice to characterise the work of lab technicians. With this characterisation in place, we can then provide an argument that lab technicians are owed moral repair by the institutions in which they work, according to what we will call ‘the duty of special concern’. Our discussion of objections to our argument clarifies the details of what is required by this duty.

## 2 The First ‘Easy’ Answer: No Duty to Offer Moral Repair

The first easy answer to the question of whether we ought to offer moral repair to individuals is ‘no’, there is no obligation in cases where individuals have committed some wrong action even if they are doing so under a degree of institutional pressure (so long as that institutional pressure is not a forcing pressure).

To explore this answer, consider a case from the moral injury literature; a soldier kills an innocent civilian while acting under some form of institutional pressure (acting under orders, e.g.) and develops moral injury. Psychologists and others then spend considerable effort determining how to effect moral repair for this morally injured soldier. However, surely this is the wrong focus. Our focus should be on assisting those harmed by this soldier. Our response to the soldier and the institution that pressured them to kill should be one of blame. Instead, we are offering to provide help to the soldier in the form of psychological support services. Appealing to the presence of institutional pressures (‘I was acting under orders!’) should not alter our judgement of the moral status of the individual's actions in cases such as these. When institution and individual are jointly responsible for an immoral act, excuses like this only serve to remind us that the blame is not shifted to the institution, rather it includes the institution.^2^

With laboratory technicians, a similar argument could be made. It seems to be wrong to focus on restoring the psychological health of lab technicians when they are causing laboratory animals to suffer.

If you are someone who is convinced that all animal experimentation which causes animals to suffer is wrong, perhaps you will be satisfied with this easy answer. We do not mean to denigrate it by calling it ‘easy’ but we do think that it misses the intuition that lab technicians, at least occasionally, do something immoral yet are in some sense ‘victims’.

One case concerning lab technicians where this easy answer does seem correct is with acts of cruelty towards animals. Imagine a lab technician causes an animal to suffer by performing acts not required by the experiment. Suppose further that this lab technician causes this suffering for their own pleasure but is subsequently discovered, regrets their action, and sustains a moral injury. What seems appropriate here is to punish this lab technician and if possible, offer support to the victimised animal. The lab technician may seek moral repair on their own, but their institution would not be obliged to offer it to them. However, we take cases such as this to be rare, particularly in contemporary research.^3^

## 3 The Second ‘Easy’ Answer: Offering Moral Repair as Uncontroversial Assistance

The other easy answer is that those who wrongly believe that they have committed an immoral act ought to be owed moral repair as a simple form of assistance.^4^ Whether or not the act committed is immoral is not a necessary condition for moral injury. The necessary condition is that the act is *perceived* to be immoral by the person committing it [7]. In some cases, one may believe that one has done something wrong, but they are mistaken because they lack some salient piece of information. Consider the example of someone who mistakenly believes they have offended a friend and thereby suffers moral injury. Suppose that this person comes to you, reveals how distraught they feel, but you know that the friend in question was not at all offended by the comment (and maybe even that the comment was not offensive at any rate). In such circumstances it seems right to alleviate this persons' suffering by correcting their mistaken belief.

For lab technicians, cases such as this may occur, but we expect that they will be rare and uncontroversial to resolve. Perhaps some lab technicians incorrectly believe that they have administered the wrong drug and confess their mistake to a colleague. That colleague might have double checked the procedure and know that there was no mistake. It is uncontroversial to believe that colleague should simply correct their mistaken belief. However, this case does not speak to the puzzling intuition we outlined above, since there are some cases where lab technicians develop moral injury because they actually have done something immoral yet can be considered a ‘victim’.

## 4 Imperfect Systems of Justice

Neither of the ‘easy’ answers seem to provide adequately nuanced normative guidance. Cases where lab technicians are merely mistaken about causing harm to animals would be rare, and cases where they perform something uncontroversially immoral don't address the moral complexities lab technicians face in the normal course of their work. We need a more nuanced answer that acknowledges the moral ambiguities involved. For this, we propose drawing upon Rawls' concept of *imperfect procedural justice* [18].

In *A Theory of Justice*, Rawls makes the distinction between perfect, imperfect and pure procedural justice. Pure procedural justice does not specify which outcomes are just or desirable, rather any outcome that results from correctly following procedures are acceptable. Rawls offers the example of gambling where the resulting distribution of cash is acceptable so long as no one cheats. This conception of justice does not apply to the case of animal experimentation, since we do have an independently defined criterion for desired outcomes. Specifically, we want lab technicians to only conduct ethically permissible experiments.

When procedural justice is perfect, there is an independent criterion of the just or desired outcome and a procedure that guarantees that outcome is reached if the rules are followed perfectly. Rawls gives the example of dividing a cake equally where the person to cut the cake takes the last piece.

Cases of perfect procedural justice in general are rare. In general, it is difficult to design procedures that *guarantee* an outcome, and it is difficult to design procedures that perfectly accurately determine whether the original procedure was successful [19]. So, it is very unlikely that procedures to determine the ethical permissibility of animal experiments are perfect, especially given the morally contested nature of the field.

That is, it is possible for proposed experiments to go through all procedures for determining their permissibility, and yet be incorrectly identified as permissible, which leaves imperfect procedures of justice. Rawls gives the example of the legal system to illustrate an imperfect procedure of justice, where the desired outcome is for only those individuals who have committed crimes to be declared guilty. When an imperfect procedure produces an outcome contrary to what is desired, Rawls calls this a *miscarriage of justice*. The situation of lab technicians is a system of imperfect procedural justice. An independent criterion of the desired outcome exists, but no system *guarantees* that that outcome will always be reached when its rules are perfectly followed.

We will also label cases where the imperfect procedure for determining which experiments are ethically permissible constitute miscarriages of justice. Justice can be miscarried in two ways in the case of experiments. Experiments that are ethically permissible can be rejected and experiments that are ethically impermissible can be approved. Our focus will be on the latter since, as we will go on to argue, we have duties to those who suffer from these miscarriages of justice.^5^

Both ‘easy’ cases failed to make sense of the situation of lab technicians. The puzzling intuition they fail to capture is that lab technicians sustained moral injury, yet were, in some sense, a victim. The first easy answer fails to capture the intuition that lab technicians have done something wrong and only applies to uncontroversial cases. The second easy answer acknowledges that lab technicians have done something wrong but fails to account for the overall aims of the institution. Specifically, the second easy answer assumes that the institution has unjust aims. By understanding lab technicians as operating within imperfect systems of justice, we can begin to account for this puzzling intuition. Lab technicians operate within an institution that aims to act in accordance with justice but sometimes fails to do so. Characterising lab technicians as working within imperfect systems of justice also draws our attention to two further ethically salient features. The first is that lab technicians are institutionally pressured to carry out the experiment. The second is that lab technicians bear a substantial risk of harm since they are the ones carrying out the experiment, yet they are not the ones who designed the system [5].

With the concepts of imperfect procedural justice and miscarriages of justice in place, we can now make the argument that institutions have a duty to offer moral repair to morally injured lab technicians.

## 5 The Duty for Moral Repair as Required by the Duty for Special Concern

Here we will argue that, ceteris parabis, *within a cooperative system of mutual advantage, individuals deserve special concern for their wellbeing when they willingly assume substantial risks for the benefit of others within that cooperative system*. Call this the *duty of special concern*. We will proceed by reflective equilibrium to explain the considered intuitions that are expressed by this principle. We then show how it makes sense of several cases from institutional settings and the literature on moral injury. Finally, we argue that lab technicians fulfil the criteria for being recipients of the duty for special concern, which implies in their case a duty to offer them moral repair.

The guiding intuition behind this principle is that it is unfair and uncaring to ask others to risk their wellbeing for our sake, without supporting their wellbeing when in a cooperative setting. The principle is motivated by fairness and compassion. It is motivated by fairness insofar as we are discussing a distribution of harms and benefits. More pointedly, it seems unfair to ask people to take on dangerous tasks without offering something in return. It is also motivated by compassion in that we are responding to others' vulnerability [20]. It seems uncompassionate to ask people to take on serious risks, without showing reasonable concern for their wellbeing.

To begin, this principle only arises within cooperative systems of mutual advantage. Those who take on substantial risks as an individual in a ‘state of nature’ have no others to lay the duty upon. Supposing Robinson Crusoe takes on a substantial risk. He only has himself to care for his welfare. However, were Robinson Crusoe to take on a substantial risk for other shipwrecked sailors, he could expect them to provide him with tools to help him overcome his challenge. Similarly, those who take on risks only to benefit themselves are not eligible for receiving special concern based on this principle even if they are members of a cooperative system. And those who take risks that do not benefit others, such as those pursuing pointless or harmful endeavours, cannot claim special concern.

Finally, the individual must willingly assume a *substantial* risk to be owed this duty. We take substantial risk to be a broad term that covers many potential harms. One may risk their health or even their lives for others. What counts as ‘substantial’ is understood in comparison to the ‘ordinary’ risks routinely assumed by others within the scheme of cooperation.

To show special concern for wellbeing entails adopting measures that protect another's wellbeing given the nature of the risk they are taking. As such, special concern encompasses a wide range of measures since there are many risks we can take. What is required here is to provide protections that meet or mitigate the risks people undertaking a task are reasonably expected to face and provide support if those risks eventuate as harms. For example, to care for the wellbeing of police officers, training and personal protective equipment may be provided to meet the risks specific to that job. Here we note that sometimes measures serve multiple functions. For example, training police helps them to both to perform their job safely and to perform it well. Some measures will be prophylactic since this mitigates risk (such as training) whereas others will be provided only in cases where harm has occurred (such as sustaining an injury).^6^

Let us now examine a few cases that demonstrate the intuitiveness of this principle. Consider those who take on substantial risks such as firefighters, police officers, and soldiers. Now consider how unintuitive it would be to provide office workers with body armour and tasers but not police officers. Aside from office workers having little need for body armour, this distribution would demonstrate an inappropriately high regard for the risks they assume compared to the risks police officers assume.

One may object that the duty for special concern is encompassed by the duty of care. Both require us to provide support for another's wellbeing. However, the duty of care responds to the vulnerability of others and is motivated by compassion alone, whereas the duty for special concern is motivated by both compassion and fairness. For instance, doctors have a duty of care for patients regardless of whether the patient has taken on a substantial risk for the benefit of others.

So, with both the principle of special concern and the notion of imperfect procedures of justice in place, we can now articulate a moral justification for why lab technicians are owed moral repair.

Lab technicians work for the benefit of others by carrying out experimental procedures on animals. In adopting this role, lab technicians take on a substantial risk of moral injury given that in an imperfect system of justice unethical experiments will sometimes be approved. We can treat moral injury as a ‘substantial’ as opposed to an ‘ordinary’ risk given the severity of mental health outcomes described by the psychological literature and the prevalence of psychological turmoil in lab technicians [21].^7^ We have designed our institutions such that lab technicians assume a substantial risk of moral injury in their work [5]. To conform to the duty of special concern requires us to provide special measures that mitigate that risk and provide support whenever that harm does eventuate. That is, lab technicians should be owed moral repair.^8^

## 6 Imperfect Procedures of Justice Imperfectly Followed

To nuance our normative recommendations, we build on Rawls' concept of imperfect procedural justice. Recall that imperfect procedures of justice do not guarantee a desired outcome (i.e., one that satisfies an independent criterion) *even if their procedures are properly followed*. Imperfect procedures, as Rawls describes them, assume that people faithfully follow procedures and that independent criteria are definitely, as opposed to indefinitely, specified. In practice, however, people may fail to follow procedures correctly and work with vague independent criteria. Call cases where procedures are followed faithfully, ‘perfectly followed’ and those where they are not ‘imperfectly followed’. Let us chart these possibilities and note their significance for discharging the duty to offer moral repair.

Imperfect procedures that are perfectly followed are simply imperfect procedures of justice as Rawls defines them. With definitely defined independent criteria, they are imperfect procedures of justice as Rawls presents them. However, the systems for determining whether animal experiments are ethically permissible are not always perfectly followed, nor do they have a definite criterion.

We now explore the first of our proposed developments to Rawls' concept; that procedures may be perfectly or imperfectly followed.

For procedures to be perfectly followed, they must be followed faithfully. In cases of imperfect systems of justice where procedures are perfectly followed and where there is a miscarriage of justice, the duty of special concern outlined above should be discharged. That is, lab technicians should be offered moral repair.

However, agents follow procedures imperfectly when they fail to follow them faithfully. Agents may misinterpret rules, they may forget steps in procedures, they may intentionally cheat the system for personal gain and so on. We will divide cases of imperfectly followed procedures into three types. First, there are cases of undertraining where procedures are not faithfully followed because of improper training. Second, there are cases of negligence where a miscarriage occurs with properly trained staff who neglect to follow a procedure. Finally, there are cases of malpractice where procedures are manipulated for personal gain. Any of these types of imperfect following may lead to a miscarriage of justice. We do not take this to be an exhaustive list, but we do think it covers many important cases.

In cases where a miscarriage of justice occurs due to undertraining, the duty of special concern recommends both that appropriate training be provided and psychological services be offered to anyone who sustains moral injury. These measures show appropriate special concern for lab technicians.

In the case of negligence and malpractice, however, moral responsibility for the harm shifts in part or in full to the negligent or malpracticing individual. The responsibility shifts away from the institution since the lab technicians' moral injury was not the result of their procedures, rather it was the result of the decisions of the negligent or malpracticing individual.

### 6.1 Objection: Meta‐Systems

Part of discharging the duty for moral repair is to offer measures, such as counselling, when the harm of moral injury has occurred.^9^ An objection may arise that someone will be required to demonstrate that the experiment was in fact immoral or that the institution is in some way responsible for the moral injury otherwise the institution has no need to discharge their duty. By itself, the fact that a lab technician sustained a moral injury would not be sufficient evidence that the institution owes moral repair. The institution may insist that no miscarriage of justice occurred and that this is an easy case of mistaken belief, or individuals may wrongly claim they were morally injured at work and receive resources they do not deserve. What is needed is another system to determine whether the duty ought to be discharged. For brevity, call these meta‐systems. Suppose institutions adopt a ‘suspicious’ meta‐system whereby lab technicians are required to demonstrate that an experiment was immoral or that they sustained their moral injury because of their work if they want to receive moral repair. Wouldn't such a requirement be overly onerous? The morally injured lab technician would need to possess a competent understanding of what constitutes an immoral experiment to make their case. Not only would they need to deal with the stress of being morally injured, but they would also need to take on the daunting task of challenging their institution. Given the morally contested nature of animal experimentation, such challenges could become lengthy and draining debates. The risk here is that lab technicians who deserve moral repair would be denied it or even harmed further by going through this process.

Now suppose institutions adopted a ‘lenient’ meta‐system where minimal procedures are in place to determine the ethical permissibility of the experiment and it was assumed that any lab technician who claimed to be morally injured could receive moral repair. In such a system, lab technicians could claim resources from their institution to which they are not entitled.

Finally, some meta‐systems may be ‘poorly‐designed’. These may have high rates of miscarriage, be costly, slow and inefficient.

Which of these meta‐systems would be in conformity with duty? Our answer is that technically they all would be, so long as they attempt to offer moral repair, but not all meta‐systems would be equally desirable to fair‐minded persons. We assume that individuals will want to adopt the most desirable system that can reasonably be implemented. Further, we assume that individuals will want to adopt systems that are justifiable to fair‐minded persons within the institution.

Fair minded persons will understand that imperfect procedures need to balance competing values (cost, speediness, accuracy), they also know that acceptable systems cannot be perfect systems. They will take an interest in how they are impacted by the institution but also consider how others are impacted. They may accept, for example, that they are exposed to inaccuracies but are saved from lengthy investigations and costly expenditures. Ideally, fair minded people will come to these judgements under favourable conditions. For example, they should have sufficient opportunity to consider proposals and should not currently be under investigation.

Let us now provide a recommendation for how this duty should be best discharged, given certain facts about how the relevant institutions are currently organised and some reasonable desires of relevant individuals within those institutions.

We must assume that decision‐makers within these institutions will have a sense of justice and be motivated to discharge their duty to offer moral repair. We then further assume that they would reasonably desire that (1) experiments are conducted properly and (2) the institution's resources are used economically such that the institution, at minimum, remains economically viable.

With that in place, we survey some relevant prudential considerations and offer a recommendation for how institutions may implement this duty. We can assume that morally injured lab technicians are less effective workers than if they were morally uninjured. We can also reasonably infer that moral injury is relatively widespread among lab technicians [21]. That it is reasonable to think that moral injury is a normal risk in the work of lab technicians given the morally contestable nature of their work; that harming animals is morally charged and that the system for determining the permissibility of experiments is imperfect. Next, we assume that the costs of investigation would tend to be onerous. Beyond the financial and labour costs of performing the actual investigation, there would be costs to the workplace culture. Working in an institution that meets the claims for help of psychologically distressed colleagues with suspicion would hardly inspire loyalty among staff. Finally, we assume that there will be minimal free riding since what is offered is a service that is only useful if one already is morally injured.

As such, we recommend that institutions adopt ‘lenient’ systems wherein the assumption is that those asking for moral repair are making genuine claims. That is, they are morally injured and that moral injury has resulted from the course of their work. A simple meeting with a manager, Human Resources Representative or some other appropriate person may suffice to ascertain this. Businesses, however, could opt for more ‘suspicious’ systems, but doing so would be inadvisable given the salient prudential features outlined above.

### 6.2 Objection: The Relevant System of Cooperation

So far, we have assumed that the relevant institution that should discharge the duty to the lab technician is the institution that conducts the research. Our recommendations have been designed with research institutions in mind. However, an objection may run that systems of mutual advantage extend beyond the boundaries of research institutions. In universities, for example, animal experimentation benefits from tax‐payer dollars and benefits society through research output. Given the importance of mutual advantage to grounding duties of distributive justice we may think that someone other than, or in addition to, the institutions that conduct animal experiments, should discharge the duty.

We regard this objection as having merit, but we consider that it does not undermine our central claim that institutions that conduct animal research have a duty to offer moral repair. Aside from being a part of the system of mutual advantage, those institutions that conduct animal experiments design the systems that regulate the distribution of costs and benefits, and are well‐placed to develop systems that offer moral repair.

However, systems currently exist whereby civil institutions can receive subsidies for offering services such as this. For example, Australian states already provide businesses with free mental health resources and training. While we will not pursue this suggestion further here due to space constraints, we would prima facie support extending this duty to wider society.

## 7 Conclusion

At first blush, offering support to those who have committed an immoral act is puzzling. Intuitively, we would normally blame those who have done wrong and offer support to those who are victims of that wrong. The work of lab technicians offers an ethically puzzling case whereby we intuitively believe that we should offer support to both the person who committed the wrong (the lab technician) and the victim of the wrong (experimental animals). By drawing on Rawls' notion of imperfect procedures of justice and proposing a duty of special concern, this puzzle can be explained.

The duty of special concern requires us to provide special care for the welfare of those who willingly take on substantial risks for the benefit of others within a cooperative system. Lab technicians are people who take on substantial risks. They risk becoming morally injured as their job requires them to sometimes assist with conducting experiments that cause unjustifiable harm to animals. Yet they are not immoral people working for immoral organisations. They work for organisations that aim to produce moral outcomes but sometimes fail to do so. That is, they work in imperfect systems of justice.

The duty of special concern would be violated if institutions did not put systems in place to offer lab technicians moral repair. Failing this duty is both unfair and uncaring. We surveyed potential systems that organisations could put in place and suggested that ‘lenient’ systems would be both in conformity with the duty and advisable given reasonable prudential concerns.

## Conflicts of Interest

The authors declare no conflicts of interest.

## Data Availability

The authors have nothing to report.

## Endnotes

1 As noted in Shay's and Litz and colleagues' definitions, moral injury can also be caused by (i) witnessing acts that violate moral principles and, (ii) permissible yet morally regrettable acts such as killing and harming [2]. The focus in the main body of this text will be on moral injury that arises from impermissible acts since, as we will soon discuss, this yields an ethical puzzle. Our argument for offering moral repair to lab technicians extends to cases of moral injury that arise from causes other than committing an impermissible act, but including these cases in the main text becomes cumbersome. Instead, we shall note where these permissible‐yet‐morally‐regrettable cases are covered by our arguments in the endnotes (see also endnote 8).

2 However, this may alter the degree of blameworthiness for actions. Those acting under greater institutional pressure may have done wrong but be less blameworthy than those who did wrong under minimal institutional pressure, since the latter show a greater willingness to act immorally.

3 One infamous historical case from 1984 is that of University of Pennsylvania's Head Injury Clinic in Philadelphia where primates were abused and researchers were captured on tape making fun of the animals.

4 One may want to bolster this argument with reference to a duty of easy assistance but doing so is unnecessary for our present purposes.

5 Henceforth, ‘miscarriage of justice’ refers to the latter kind of miscarriage.

6 To be accurate, the duty of moral repair should have the more cumbersome title of ‘The duty to provide mental healthcare services aimed at preventing moral injury and achieving moral repair when moral injury has been sustained in the course of work’.

7 This duty would also extend to others in animal research institutions such as Researchers and Animal Welfare Officers if they are exposed to a substantial risk of moral injury (or indeed, any substantial risk). However, there is currently an insufficient academic literature on the psychological effects of animal research on these roles. If an institution had reason to think that another role was also subject to substantial risk, then they would be required to discharge special concern.

8 In endnote 1, we noted that our focus is on lab technicians who sustain moral injury from committing an impermissible act. Permissible yet morally regrettable acts that cause moral injury are also covered by our argument since lab technicians are the ones assigned to conduct acts such as killing animals in ethical experiments, which bears the risk of moral injury.

9 Another part of discharging the duty is taking reasonable preventative measures. We have alluded to adequate training but, for reasons of space, our discussion of preventative measures is limited. We would like to thank an anonymous reviewer for raising an interesting question related to prevention. Namely, whether institutions should selectively hire staff whose morality aligns with animal research. While selective hiring does cohere with the duty of special concern, there may be other ethical considerations that make this specific proposal more complex such as the risk of having an ethically homogenous workforce that experiments on vulnerable animals.
